# Unused and Expired Medications Disposal Practices among the General Public in Selangor, Malaysia

**DOI:** 10.3390/pharmacy8040196

**Published:** 2020-10-23

**Authors:** Mohamed Azmi Hassali, Sadia Shakeel

**Affiliations:** 1 Discipline of Social and Administrative Pharmacy, School of Pharmaceutical Sciences, University Sains Malaysia (USM), Minden 11800, Malaysia; sadiashakeel@usm.my; 2 Faculty of Pharmaceutical Sciences, Dow University of Health Sciences, Karachi 74200, Pakistan

**Keywords:** medication wastage, awareness, attitude, medication disposal practices, Malaysia

## Abstract

The appropriate disposal practice of unused and expired medications has become a global challenge that has caught the attention of health policymakers, pharmaceutical organizations, healthcare professionals, and the wider community. The current study aimed to evaluate the awareness, attitudes, and behaviors relating to the disposal practice of unused and expired medications and medication wastage issues among the general public in Selangor, Malaysia. The quantitative, cross-sectional study was conducted using a pre-validated structured survey form. Among the approached individuals, 426 showed their willingness to participate in the study. More than 80% of the study population reported being aware of the medication wastage issue and its impact on patients and the economy. The respondents with a higher level of education (OR = 1.85; 95% CI = 1.18–2.52; *p* < 0.003) were more likely to be cognizant of the detrimental consequences of inappropriate waste disposal. The female respondents were more likely to report comprehending that the availability of free healthcare resources is contributing to medication waste (OR = 1.33, 95% CI = 1.015–2.34; *p* < 0.005). The majority of respondents reported throwing away unused medications (202; 47.4%) and expired medications (362; 84.9%) in the garbage. The respondents believed that the provision of appropriate directions by healthcare professionals (312; 73.2%) and prescribing/dispensing medications in quantities for the duration that ensures patient adherence (114; 26.7%) could minimize medication wastage. The Ministry of Health (258; 60.5%), pharmaceutical organizations (212; 49.7%), and pharmacists (193; 45.3%) were the respondents’ perceived responsible sources of information. The current findings reported that respondents were familiar that inappropriate practices of medication wastage might have harmful consequences. However, a gap exists between their awareness and practice, and the disposal approaches practiced by the respondents were generally not appropriate.

## 1. Introduction

A major waste category, whose management is a complex and troublesome issue with regards to the worldwide population growth rate, is medication wastage. As per the World Health Organization (WHO), medical waste is characterized as “waste generated in the diagnosis, treatment or immunization of humans or animals” [1]. As medical waste is possibly hazardous and contaminating, its inappropriate disposal practices represent a risk to general wellbeing and to the environment [2].

Medications represent the major segment of medical waste. Medicinal or pharmaceutical waste or unused or expired medications comprise of articles proposed for the prevention, diagnosis, and treatment of diseases in human beings or animals. This type of waste has been broadly studied in recent years [3,4,5]. There are presently more than 3000 active pharmaceutical ingredients (APIs) on the EU market that are approved, and more than 4000 are available around the world. Furthermore, universal evolution in the awareness of health-seeking behavior among individuals has given rise to an increase in the consumption of medications. The annual overall medication utilization surpasses 1,000,000 tons and is consistently expanding equally for prescription and over-the-counter (OTC) medications [1]. However, due to several reasons, patients may not consume all of the medications prescribed or dispensed to them [3]. The main reasons include relief of the symptoms, dosage alterations, forgetfulness, adverse effect intolerance, and medications reaching their expiry date. Furthermore, sometimes general practitioners may prescribe needlessly large quantities, and pharmacists may likewise dispense a larger amount of medication as manufacturers’ package sizes may exceed the quantity needed for treatment. Hence, all of the pharmaceutical products are not consumed and large quantities remain unused or become expired [4].

Inappropriate disposal of medication poses a significant environmental risk, and continuous environmental exposure to medicinal products can have dangerous effects [2]. Kusturica et al. [6] investigated and compared the association between disposal of unused medications and awareness of environmental issues in different nations over the globe from 2005 to 2015, utilizing the peer-reviewed literature, finding that there were no set policies or standards for the disposal of unwanted medications. This exploration additionally found that unused medications were often discarded by being flushed down the toilet or discharged into watercourses, raising genuine environmental concerns. The residues of pharmaceutical waste are openly dumped in soil; later, they reach water supplies and their decomposition derivatives influence the natural life. For instance, the continuing exposure to specific estrogens (such as 17-ethinylestradiol) found in contraceptive pills has directed the feminization of male rats [7]. Evidence revealed that antibiotics present in the environment could lead to antibiotic resistance. Though, human wellbeing concerns are perturbing to a lesser extent, as the concentrations of antibiotics in drinking water are considerably lesser than the usually suggested doses in treatment [6]. However, the continuing summation of a specific form of a chemical has irrational effects in the body and the hazards accompanying these exposures should be considered. Besides, inappropriate medication disposal might impose an economic burden on patients along with the healthcare system [8]. Unused medications can be a source of healthcare resource depletion. It has been anticipated that billions of dollars’ worth of unused medication are waste each year [2]. Moreover, the storing of medications at home encourages the practice of self-medication that may cause adverse consequences and offers opportunities for abuse and misuse if accidentally ingested [9].

It is important that patients dispose of these medications appropriately, for example, by returning them to pharmacies or chemical waste depots. Hence, the appropriate disposal practices of medication wastage have become a challenge globally that has caught the attention of healthcare professionals, pharmaceutical companies, policymakers, and the wider community [4]. Various nations have established waste medication recycling mechanisms as the safe disposal of unused medications, especially by users, is of great importance. Many industrialized countries have programs for the return and disposal of unwanted medications, which has the full support of the pharmaceutical industries and the government [10]. For instance, community pharmacies in the United States, France, Italy, and Sweden are liable for collecting the disposed of medications, and pharmacists likewise provide information about safe disposal practices of unwanted medications. However, in some countries, systems or programs that promote safe disposal practices for unused medications remain limited [11].

Malaysia is an Asian nation with a population of 32.7 million [12]. The utilization of pharmaceutical medications is common, including modern and traditional medications. Medication wastage could be avoided by assuring that medications are properly utilized and prescribed just when required, and are used as recommended. The receiving stations for unused medications need to be built up across Malaysia, medications return policies and guidelines need to be planned, and the unrestricted disposal practices of unused and expired medications by people should be avoided [13].

Given the exploration outlined above, this study expected to:

- Comprehend the awareness and attitude towards medication wastage issues among the general public in Selangor, Malaysia.
- Identify the behaviors by which individuals from the general population discard unused and expired medications, alongside their familiarity with related issue.
- Know the major reasons for keeping unused medications at home.
- Recognize the general public’s perceived responsible sources of information for reducing the medication wastage issue in Malaysia.

## 2. Materials and Methods

### 2.1. Study Design

The quantitative, cross-sectional study was conducted by face-to-face interviews using a pre-validated structured survey form in Selangor, Malaysia from September to December 2019.

### 2.2. Study Population

The study population included students, private and public sector employees, and housewives; who were over 18 years of age, residents of Selangor, irrespective of ethnic origin or employment status. Individuals visiting public places, such as the shopping malls, night markets, universities, restaurants, and other recreational areas in the Cheras area, were approached and invited to join the study. Cheras is a town and a district, straddling both the Federal Territory of Kuala Lumpur and the Hulu Langat District in Selangor state, Malaysia. The township is located in the south-east of downtown Kuala Lumpur. Cheras is also adjacent to Ampang to the north and Kajang to the south, both of which are the major towns within the metropolitan area of Greater Kuala Lumpur. Upon approaching potential respondents, they were provided with an outline of the study objective and their time needed for the study. There were no incentives presented to the respondents for participation in the study.

### 2.3. Sample Size Calculation

The Raosoft sample size calculator was used to calculate the required sample size by utilizing a confidence level of 95%, with a 5% margin error and a 50% chance of respondents agreeing to take part in the study [14]. The minimum required sample size was estimated to be *n* = 385. The approach of convenience sampling was used for the study.

### 2.4. Inclusion and Exclusion Criteria

The respondents were considered eligible to be included if they: were above 18 years of age; understood English or Malay language; residents of Selangor, Malaysia; agree to contribute to the study by signing the written informed consent form. It is pertinent to mention that neither the interviewer nor any of the researchers had any personal or professional association with any of the interviewees. Participation in the study was voluntary and those who did not meet any of these criteria were excluded from the study.

### 2.5. Study Tool

A pre-validated structured survey form was developed after a broad literature review [9,10,11,12]. Along with the demographic information, the survey form included 22 close-ended items to evaluate respondents’ awareness, attitudes, and behaviors relating to the disposal practice of unused and expired medications and medication wastage issues in Malaysia. On completing the content validity, the survey form was pre-tested in a smaller sample of the general public (*n* = 30), to evaluate the transparency and clarity of the question items (face validity). The internal reliability testing was calculated using Cronbach’s alpha and the value for reliability was found to be 0.896, which is acceptable to accomplish the goals of the present study. A slight modification was needed after the pilot testing. The respondents who participated in the pilot testing were not included in the final study. The survey form was used in two languages (Malay and English) to make it more understandable. The face-to-face interview technique was used for completing the survey. The respondents were given a brief description of the purpose of the study in the first section of the survey form. Informed consent was attached on the 2nd page in English and Malay language. Besides, the questionnaire consisted of 3 sections: personal information; twelve questions regarding their awareness and attitude towards the medication wastage issue in Malaysia; and ten questions were regarding their behaviors of keeping unused or expired medications at home, the disposal practice of such medications, their perceived responsible sources of information, and their perceived method/approach of reducing the medication wastage issue in Malaysia.

### 2.6. Ethical Considerations

Ethical approval was taken from the Ethics Committee, School of Pharmaceutical Sciences, USM (PPSF HECU 18/01). Written informed consent was filled by each respondent before participation, and confidentiality of the information gathered was maintained by using the respondent’s codes to label the data instead of using their names.

### 2.7. Data Analysis

The data analysis was done by using Statistical Package for the Social Sciences^®^ (SPSS) for Windows version 24.0 (IBM Corporation, Armonk, NY, USA) [15]. The items in the questionnaires were presented in frequencies and percentages. Chi-squared test (χ^2^) or Fisher’s exact test was conducted as appropriate to determine whether an association existed between the dependent and independent variables, considering a *p*-value < 0.05 as statistically significant.

## 3. Results

### 3.1. Respondents’ Demographic Information and Practice of Procuring Medications

Among the approached 600 individuals, 426 showed their willingness to participate in the study. Hence, the response rate of the present study was 71%. A large proportion of the respondents (269; 63.1%) were females. Most of the respondents were Malay (378; 88.7%), followed by Chinese (32; 7.5%). More than half of the respondents were bachelor’s degree holders (220; 51.6%) (Table 1). As regards the methods of buying medications, 339 (79.5%) respondents bought medications on prescription and 82 (19.2%) procured medications over-the-counter (OTC). The major classes of medications that were purchased included antibiotics (207; 48.5%) followed by painkillers/non-steroidal anti-inflammatory drugs (NSAIDS) (101; 23.7%). The majority (415; 97.4%) of respondents reported that they check the expiry date of the medications before buying.

### 3.2. Respondents’ Awareness and Attitude towards Medication Wastage

More than 80% of the study population reported being aware of the medication wastage issue in Malaysia and its impact on patients and the economy (Table 2). The respondents (349; 81.9%) knew that inappropriate medication disposal practices might have harmful consequences on both the health and the environment. The respondents with a higher level of education (OR = 1.85; 95% CI = 1.18–2.52; *p* < 0.003) were more likely to be cognizant of the detrimental consequences. More than 80% of respondents believed that the Ministry of Health and healthcare professionals could do more to reduce medication wastage. The male respondents were more likely to consider the role of healthcare professionals in combating the issue (OR = 1.157, 95% CI = 1.002–1.31; *p* < 0.001). More than 45% of respondents opined that access to free healthcare resources is contributing to medication wastage. The female respondents were more likely to report comprehending the availability of free healthcare resources and the issue of medication waste (OR = 1.33, 95% CI = 1.015–2.34; *p* < 0.005). Female respondents (OR = 1.2; 95% CI = 0.46–1.94; *p* = 0.0001) with a higher level of education (OR = 2.7; 95% CI = 1.01–4.3; *p* = 0.004) were found to be more confident in their ability to play a role in reducing the issue of medication wastage.

### 3.3. Respondents’ Practices of Discarding Unused and Expired Medications

**Figure 1:**
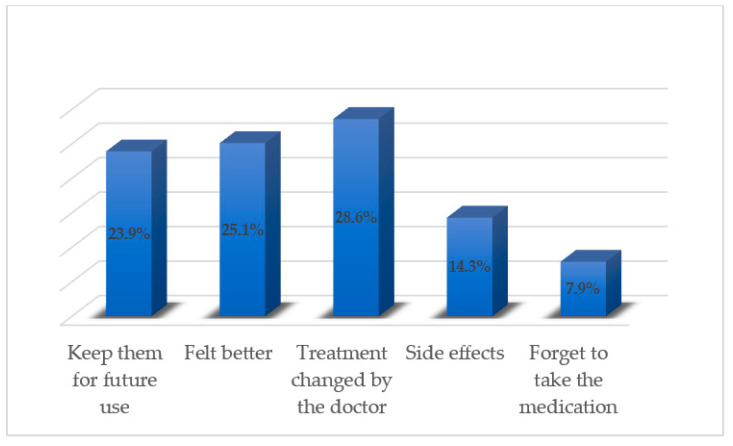
Respondents’ major reported reasons for keeping unused medications at home.

Table 3 depicted the practice of discarding unused and expired medications by the respondents. The respondents (301; 70.6%) stated to have some amount of bought medications that remain unused in their homes. The majority of respondents reported throwing away unused medications (202; 47.4%) and expired medications (362; 84.9%) in the garbage. The major reported reasons by the respondents for keeping unused medications at home were: they keep them for future use (102; 23.9%), they felt better (107; 25.1%), their treatment was changed by the doctor (122; 28.6%), they experienced side effects (61; 14.3%), and they forget to take the medication as prescribed/directed (34; 7.9%) (Figure 1). The respondents opined that the provision of appropriate directions by healthcare professionals (312; 73.2%) and prescribing medications in quantities for the duration that ensures patient adherence (114; 26.7%) could control or minimize medication wastage.

### 3.4. Respondents’ Perceived Responsible Sources of Information

**Figure 2:**
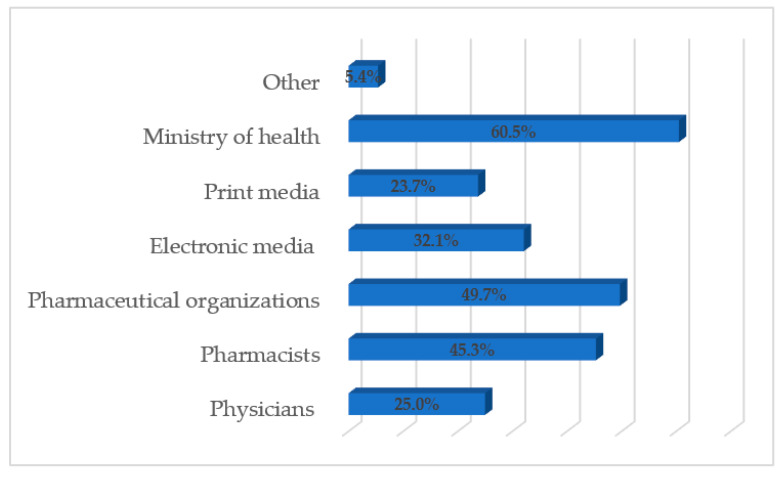
Respondents’ perceived responsible sources of information.

The Ministry of Health (258; 60.5%), pharmaceutical organizations (212; 49.7%), and pharmacists (193; 45.3%) were the respondents’ perceived responsible person/organization for creating awareness for the proper disposal of expired and unused medications (Figure 2).

## 4. Discussion

Presently, medication disposal and waste management is an important subject gripping attention, as it has been comprehended that inappropriate disposal could contaminate the surrounding areas and pose a hazard to air, water, the food chain, harm animals/livestock, and even agricultural products [6]. The appropriate collection and disposal of expired and unused medications through well-planned programs have implications for ensuring community safety and protecting the natural environment [16]. Therefore, studies have been directed all over the world around this matter to find strategic solutions [16,17,18]. In the current study, more than 80% of the study population reported being aware of the medication wastage issue in Malaysia, its impact on patients and the economy, and knew about its harmful consequences on both the health and environment. Similar findings were reported by another study in which the majority of respondents were aware of the dangerous health and environmental effects of inappropriate disposal of expired and unused medications [7,16]. The contamination of medications in water, even in a small quantity, can be life-threatening for aquatic creatures. Because of the active ingredients in medications, they can be harmful to the surrounding areas [17].

The current findings reported that around 80% of respondents bought medications on prescription and 19.2% procured medications OTC. It has been revealed that the wastage of OTC medications is more than prescribed medications [19]. Around 98% of the respondents reported that they check the expiry date of the medication before buying. A similar outcome has been reported by another study [19]. Though, in contrast, many respondents in an Indian study did not have awareness of the expiry date of medications [20]. The majority of respondents reported throwing away unused medications (47.4%) and expired medications (84.9%) in the garbage. Parallel findings were reported in another study, in which most respondents claimed to throw unwanted medications in the household garbage [21]. One study found that the most favored methods of disposing of unwanted medications was to throw them in bins and flush them in toilets [4]. A study showed that housewives in the Korean city of Busan used the standard garbage bag to dispose of expired and unused medications [22]. The correct way to dispose of expired and unused medication is to drop off the medications at a medication take-back site, or program, promptly [23]. In case it is not possible, then the next best option is to flush in the toilet/drain immediately; this is the best practice for liquid medications [24]. However, if the medications are not harmful then they can be thrown in the garbage [25]. It is recommended to mix the expired medications (without crushing capsules or tablets) with an indigestible material such as cat litter, dirt, or used coffee grounds. Then, place the mixture in a container such as a sealed plastic bag and throw it in the household garbage after removing all the personal information on the label of the empty medication packaging or pill bottles [26]. In the present study, only 30.2% of respondents crush tablets before discarding; 16.4% dilute the medication with water, whereas around half of the respondents discard the expired medications as it is. Several studies showed that not only was the practice of disposing of unused and expired medications different, but participants were not cognizant of the appropriate approaches and they kept the medication at home because they were unsure what to do with it [27,28]. In the present study, 301 (70.6%) of the respondents stated to have some quantity of purchased medications that remain unused in their home that could be the basis of possible health threats. Another study reported that half of the respondents reserved unused medications at home until they became expired [29]. It is generally observed that the majority of buyers do not know what to do with medications that remain unutilized at homes and their presence might become a source for irrational medication use and unintended or accidental poisoning [30]. A study reported that the most common reason for having unused medications was “in case they are needed later” [31].

The respondents opined that the provision of appropriate directions by healthcare professionals (73.2%) and prescribing medications in quantities for the duration that ensures patient adherence (26.7%) could control or minimize medication wastage. It is well-recognized that appropriate prescribing and dispensing activities are the major stages for reducing medication waste and may contribute significantly to combating the issue [32]. The government needs to focus on medications that are available free of charge in public hospitals, as the freely available medications are associated with increased medication waste. This result is worthy of the development of national policies regarding medication supply and targeted medication waste [33]. In the current study, the Ministry of Health, pharmaceutical organizations, and pharmacists were the respondents’ perceived responsible person/organization to create awareness for proper medication disposal. Another study’s outcomes propose that the government, pharmacists, and pharmaceutical industries were considered accountable for creating awareness towards the issue [30]. It is significant to consult a prescriber or pharmacist for dealing with unused medications as there is no guideline at present in Malaysia regarding the disposal of medications by the public. It is evident that healthcare professionals can teach the patients/caregivers medication disposal in a better way; hence, leveraging their understanding through continuous medical education and training courses is of significance [33,34,35]. Being a drug expert, pharmacists are in the forefront of tackling issues of prudently disposing of medications. They must be aware of their community’s medication disposal activities and be capable of recommending appropriate practices to their patients [8,36].

The study limitations include the cross-sectional nature of the study design that prevents us from representing causal inferences about the association between the selected covariates and outcome variables over a period. The sample size of the study was small to depict a clear picture of the entire Selangor population; hence, the findings of the current study are not generalizable to all of Malaysia. Therefore, further studies are required to validate the study outcomes. There is a predominance of females and younger respondents in the sample, so it seems unlikely to be representative of the whole population. Furthermore, the outcomes of the study were based on self-reported information that depends on the reliability and recalls the aptitude of the respondents.

## 5. Conclusions

The current findings reported that respondents knew that inappropriate practices of medication wastage might have harmful consequences on both the health and the environment. However, a gap exists between their awareness and practice of disposing of medications. To promote the enduring discussion on safe disposal practices of medication, the government should come forward to increase awareness through broad media campaigns and develop cost-effective medication waste management programs. It is likewise imperative to focus on medication waste reduction activities by healthcare professionals. Furthermore, it is essential to establish a national policy and legal outline and train staff for the successful disposing of pharmaceutical products waste.

## Figures and Tables

**Table 1:** Characteristics of study population and practice of procuring medications, *n* (%).

Age (Years)	
18–24	198 (46.4)
25–31	88 (20.6)
32 and above	140 (32.8)
**Gender**	
Male	157 (36.8)
Female	269 (63.1)
**Ethnic group**	
Malay	378 (88.7)
Chinese	32 (7.5)
Indian	6 (1.4)
Others	10 (2.3)
**Level of education**	
College/Pre-university/diploma	141 (33.0)
Graduate	220 (51.6)
Postgraduate	65 (15.2)
**Marital status**	
Single	254 (59.6)
Married	172 (40.3)
Method of purchasing medications	
Bought on prescription	339 (79.5)
Bought over-the-counter	82 (19.2)
Received from colleague/friend	5 (1.1)
**Major classes of medications procured**	
Pain killers/NSAIDs	101 (23.7)
Antibiotics	207 (48.5)
Antihypertensive	51 (11.9)
Antidiabetic	20 (4.6)
OTC antihistamines	34 (7.9)
Multi-vitamins and other supplements	13 (3.0)
**Do you check expiry date of the medications before buying?**	
Yes	415 (97.4)
No	11 (2.5)

**Table 2:** Respondents’ awareness and attitude towards medication wastage, *n* (%).

Statements	Strongly Agree/Agree	Neither Agree/Nor Disagree	Strongly Disagree/Disagree
I am aware of the medication wastage issue in Malaysia	349 (81.9)	57 (13.3)	20 (4.6)
I am aware of the impact of the medication wastage on patients	351 (82.3)	44 (10.3)	31 (7.2)
I am aware of the impact of the medication wastage on economy	357 (83.8)	39 (9.1)	30 (7.0)
I am aware that inappropriate practices of medication wastage may have harmful consequences on both the health and environment	349 (81.9)	57 (13.3)	20 (4.6)
I often purchase medications regularly whether or not they have run out	97 (22.7)	44 (10.3)	285 (66.9)
I often obtain free medications regularly whether or not they have run out	84 (19.7)	59 (13.8)	283 (66.4)
I often pass medications that I have bought for myself to relatives, neighbours or friends	153 (35.9)	49 (11.5)	224 (52.5)
I feel that free healthcare resources is contributing to the medication wastage	203 (47.6)	115 (26.9)	108 (25.3)
I feel that healthcare professionals are responsible for the issue of medication wastage	218 (51.1)	92 (21.5)	116 (27.2)
I feel confident in my ability to reduce medication wastage	236 (55.3)	151 (35.4)	39 (9.1)
I think that healthcare professionals could do more to reduce medication wastage	351 (82.3)	61 (14.3)	14 (3.2)
I think that Ministry of Health could do more to reduce medication wastage	347 (81.4)	69 (16.1)	10 (2.3)

**Table 3:** Respondents’ practices of discarding unused and expired medications.

Questions		Responses *n* (%)
Did any amount of bought medication remain unused at your home?	Yes	301 (70.6)
	No	125 (29.3)
What do you do with the unused medications?	Discard in household garbage	202 (47.4)
	Donate to hospital/charity	31 (7.2)
	Gives to friend or relative	49 (11.5)
	Return to pharmacies	35 (8.2)
	Keep at home until expired	69 (16.1)
	Flush unused medications in toilet or sink	25 (5.8)
	Don’t know what to do	15 (3.5)
What do you do with the expired medications?	Discard in household garbage	362 (84.9)
	Flush expired medications in toilet or sink	53 (12.4)
	Return to Pharmacies	5 (1.1)
	Don’t know what to do	6 (1.4)
Approach of discarding expired medications	Crush before disposal	129 (30.2)
	Dilute the medication with water	70 (16.4)
	Discard as it is	207 (48.5)
	I don’t know what to do	20 (4.6)
